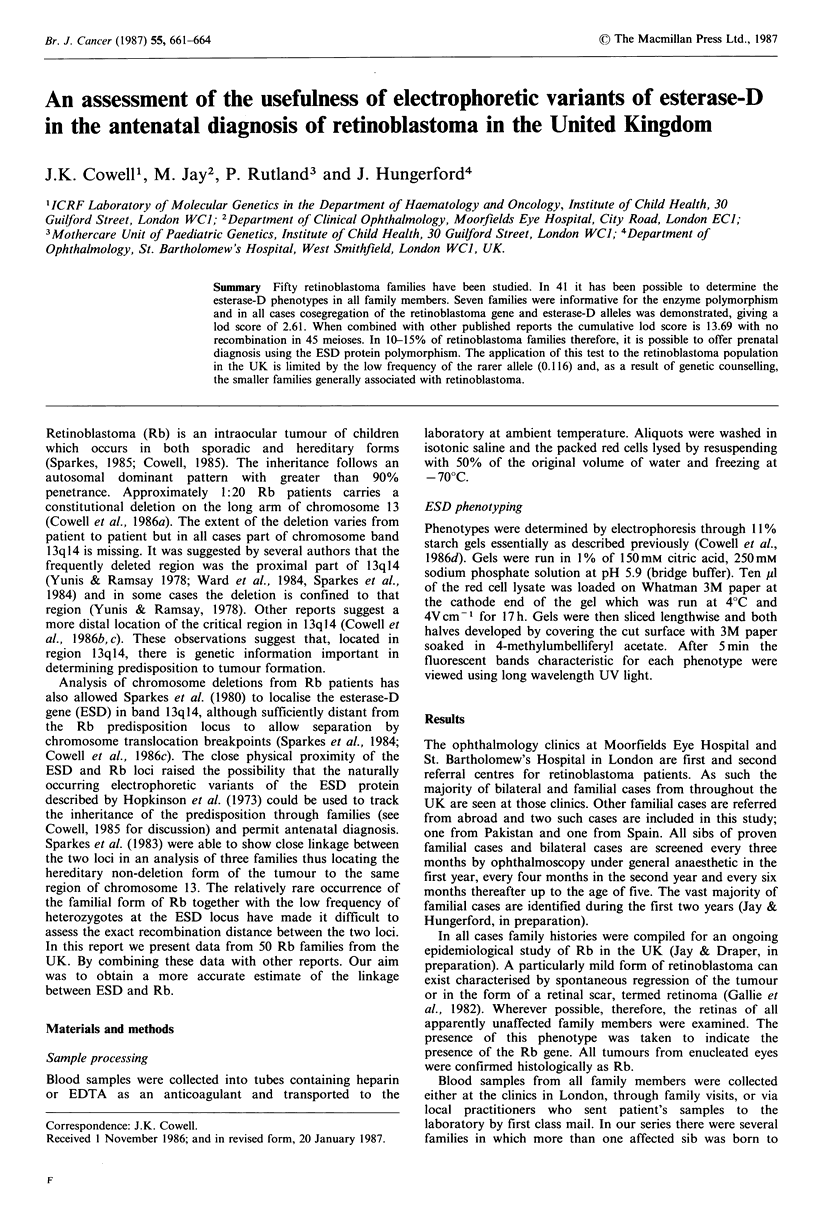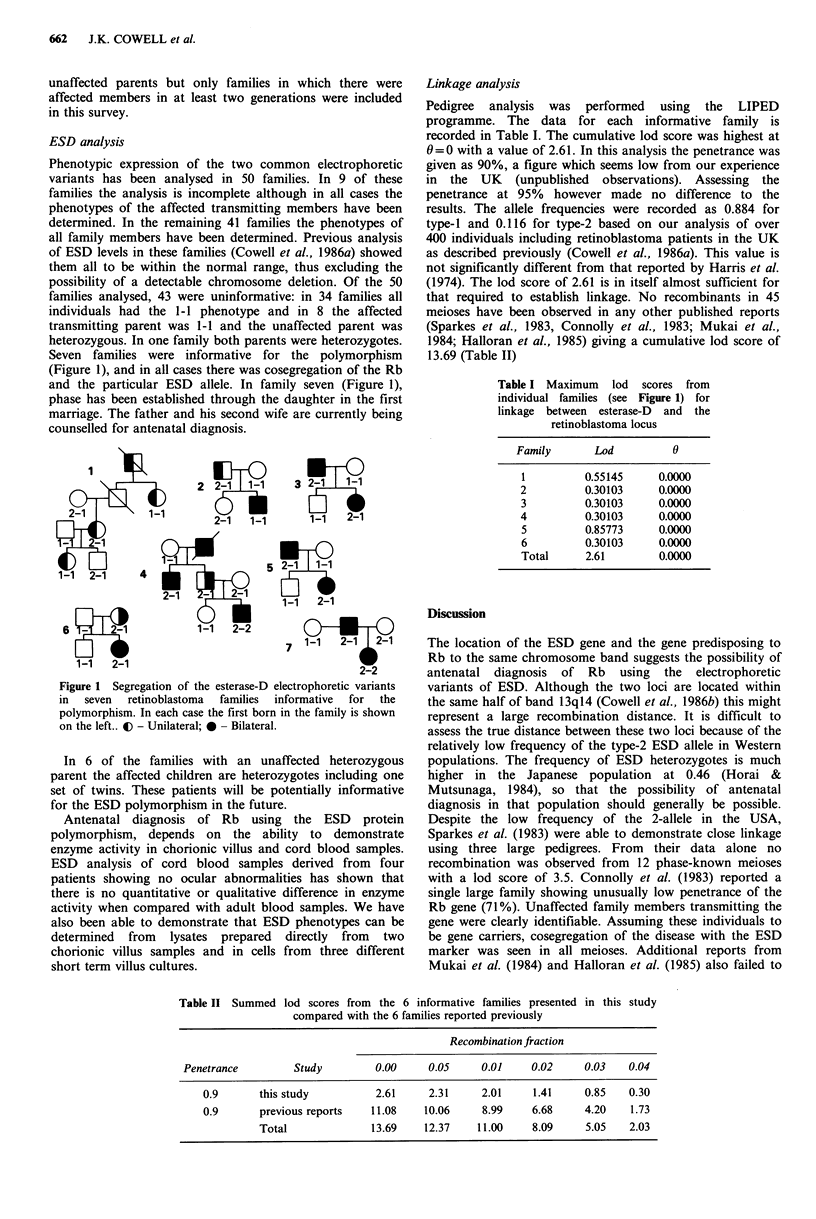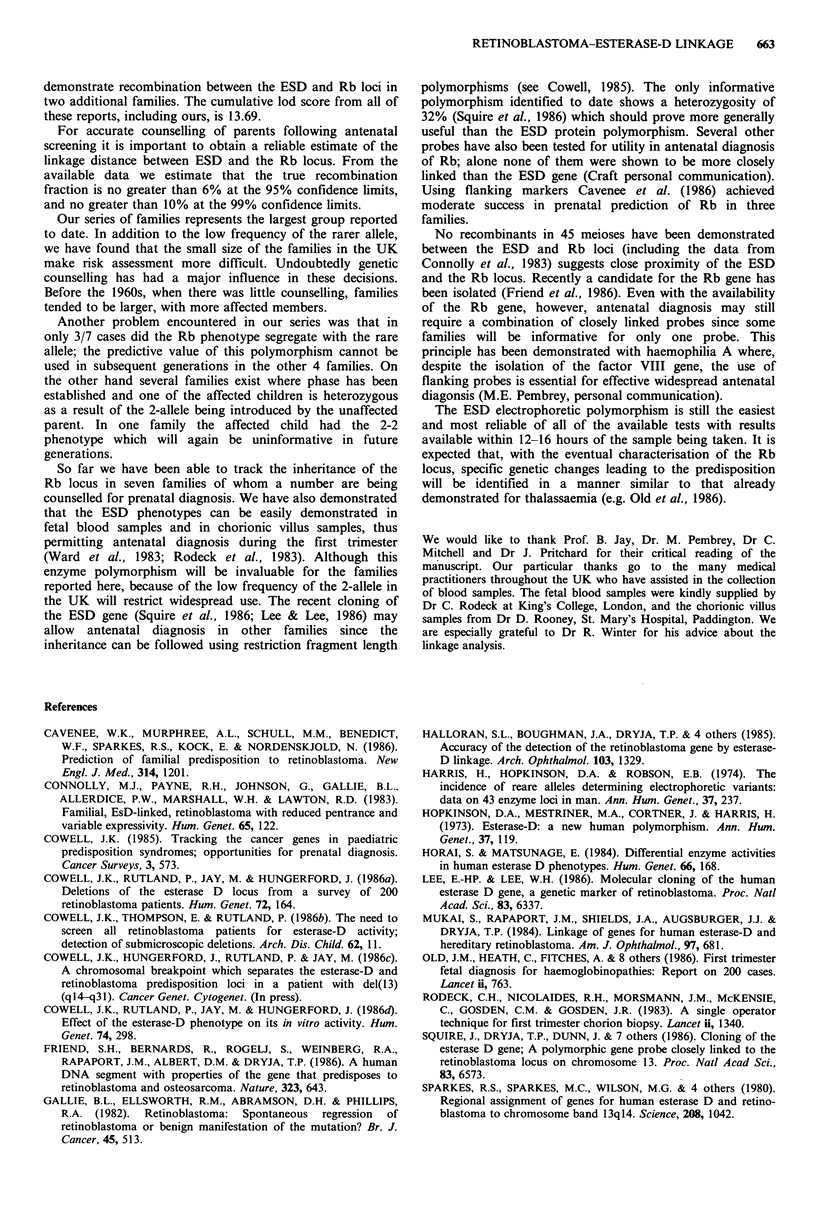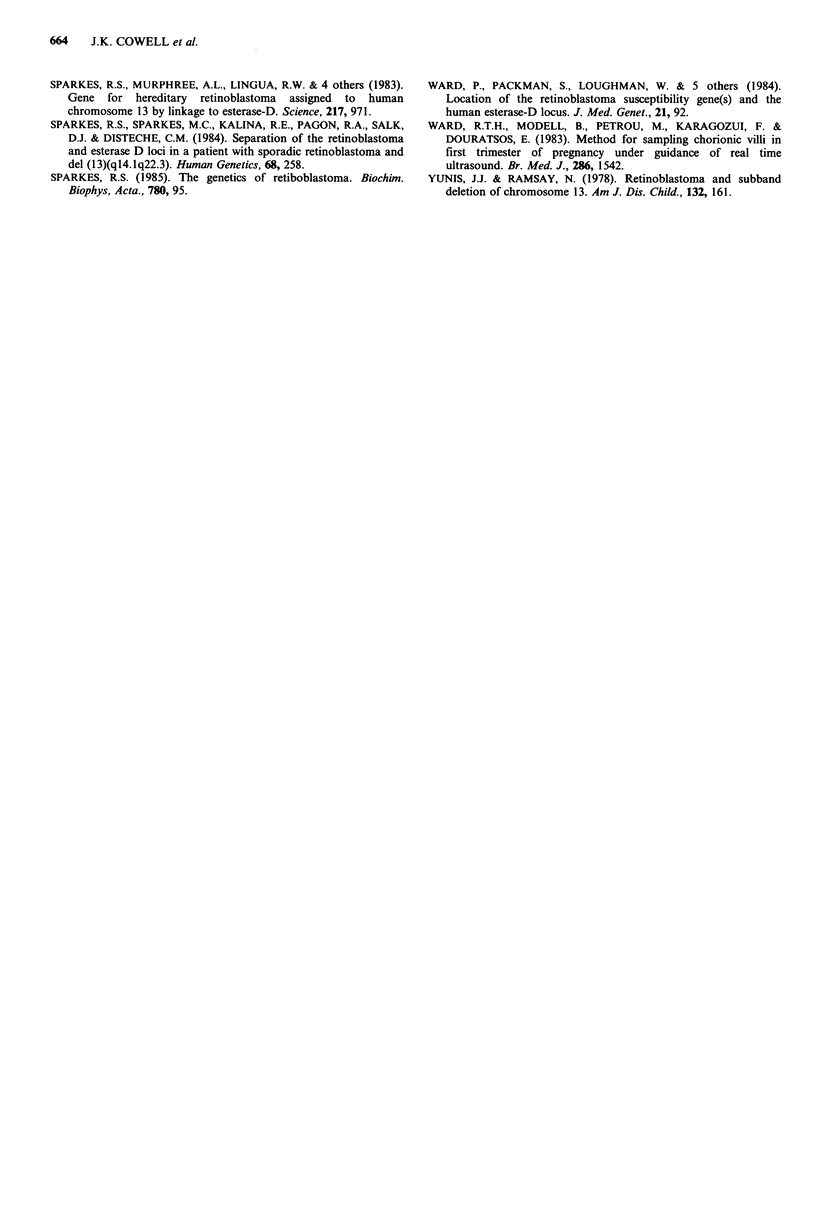# An assessment of the usefulness of electrophoretic variants of esterase-D in the antenatal diagnosis of retinoblastoma in the United Kingdom.

**DOI:** 10.1038/bjc.1987.135

**Published:** 1987-06

**Authors:** J. K. Cowell, M. Jay, P. Rutland, J. Hungerford

## Abstract

Fifty retinoblastoma families have been studied. In 41 it has been possible to determine the esterase-D phenotypes in all family members. Seven families were informative for the enzyme polymorphism and in all cases cosegregation of the retinoblastoma gene and esterase-D alleles was demonstrated, giving a lod score of 2.61. When combined with other published reports the cumulative lod score is 13.69 with no recombination in 45 meioses. In 10-15% of retinoblastoma families therefore, it is possible to offer prenatal diagnosis using the ESD protein polymorphism. The application of this test to the retinoblastoma population in the UK is limited by the low frequency of the rarer allele (0.116) and, as a result of genetic counseling, the smaller families generally associated with retinoblastoma.


Br. J. Cancer (1987) 55, 661–664 © The Macmillan Press Ltd., 1987

### An assessment of the usefulness of electrophoretic variants of esterase-D in the antenatal diagnosis of retinoblastoma in the United Kingdom

J.K. Cowell[1], M. Jay[2], P. Rutland[3] and J. Hungerford[4]

[1]*ICRF Laboratory of Molecular Genetics in the Department of Haematology and Oncology, Institute of Child Health, 30 Guilford Street, London WC1;* [2]*Department of Clinical Ophthalmology, Moorfields Eye Hospital, City Road, London EC1;* [3]*Mothercare Unit of Paediatric Genetics, Institute of Child Health, 30 Guilford Street, London WC1;* [4]*Department of Ophthalmology, St. Bartholomew's Hospital, West Smithfield, London WC1, UK.*

**Summary** Fifty retinoblastoma families have been studied. In 41 it has been possible to determine the esterase-D phenotypes in all family members. Seven families were informative for the enzyme polymorphism and in all cases cosegregation of the retinoblastoma gene and esterase-D alleles was demonstrated, giving a lod score of 2.61. When combined with other published reports the cumulative lod score is 13.69 with no recombination in 45 meioses. In 10–15% of retinoblastoma families therefore, it is possible to offer prenatal diagnosis using the ESD protein polymorphism. The application of this test to the retinoblastoma population in the UK is limited by the low frequency of the rarer allele (0.116) and, as a result of genetic counselling, the smaller families generally associated with retinoblastoma.

Retinoblastoma (Rb) is an intraocular tumour of children which occurs in both sporadic and hereditary forms (Sparkes, 1985; Cowell, 1985). The inheritance follows an autosomal dominant pattern with greater than 90% penetrance. Approximately 1:20 Rb patients carries a constitutional deletion on the long arm of chromosome 13 (Cowell *et al.*, 1986*a*). The extent of the deletion varies from patient to patient but in all cases part of chromosome band 13q14 is missing. It was suggested by several authors that the frequently deleted region was the proximal part of 13q14 (Yunis & Ramsay 1978; Ward *et al.*, 1984, Sparkes *et al.*, 1984) and in some cases the deletion is confined to that region (Yunis & Ramsay, 1978). Other reports suggest a more distal location of the critical region in 13q14 (Cowell *et al.*, 1986*b, c*). These observations suggest that, located in region 13q14, there is genetic information important in determining predisposition to tumour formation.

Analysis of chromosome deletions from Rb patients has also allowed Sparkes *et al.* (1980) to localise the esterase-D gene (ESD) in band 13q14, although sufficiently distant from the Rb predisposition locus to allow separation by chromosome translocation breakpoints (Sparkes *et al.*, 1984; Cowell *et al.*, 1986*c*). The close physical proximity of the ESD and Rb loci raised the possibility that the naturally occurring electrophoretic variants of the ESD protein described by Hopkinson *et al.* (1973) could be used to track the inheritance of the predisposition through families (see Cowell, 1985 for discussion) and permit antenatal diagnosis. Sparkes *et al.* (1983) were able to show close linkage between the two loci in an analysis of three families thus locating the hereditary non-deletion form of the tumour to the same region of chromosome 13. The relatively rare occurrence of the familial form of Rb together with the low frequency of heterozygotes at the ESD locus have made it difficult to assess the exact recombination distance between the two loci. In this report we present data from 50 Rb families from the UK. By combining these data with other reports. Our aim was to obtain a more accurate estimate of the linkage between ESD and Rb.

### Materials and methods

### *Sample processing*

Blood samples were collected into tubes containing heparin or EDTA as an anticoagulant and transported to the laboratory at ambient temperature. Aliquots were washed in isotonic saline and the packed red cells lysed by resuspending with 50% of the original volume of water and freezing at −70°C.

### *ESD phenotyping*

Phenotypes were determined by electrophoresis through 11% starch gels essentially as described previously (Cowell *et al.*, 1986*d*). Gels were run in 1% of 150mM citric acid, 250mM sodium phosphate solution at pH 5.9 (bridge buffer). Ten $\mu$l of the red cell lysate was loaded on Whatman 3M paper at the cathode end of the gel which was run at 4°C and $4\,V\,cm^{-1}$ for 17h. Gels were then sliced lengthwise and both halves developed by covering the cut surface with 3M paper soaked in 4-methylumbelliferyl acetate. After 5min the fluorescent bands characteristic for each phenotype were viewed using long wavelength UV light.

### Results

The ophthalmology clinics at Moorfields Eye Hospital and St. Bartholomew's Hospital in London are first and second referral centres for retinoblastoma patients. As such the majority of bilateral and familial cases from throughout the UK are seen at those clinics. Other familial cases are referred from abroad and two such cases are included in this study; one from Pakistan and one from Spain. All sibs of proven familial cases and bilateral cases are screened every three months by ophthalmoscopy under general anaesthetic in the first year, every four months in the second year and every six months thereafter up to the age of five. The vast majority of familial cases are identified during the first two years (Jay & Hungerford, in preparation).

In all cases family histories were compiled for an ongoing epidemiological study of Rb in the UK (Jay & Draper, in preparation). A particularly mild form of retinoblastoma can exist characterised by spontaneous regression of the tumour or in the form of a retinal scar, termed retinoma (Gallie *et al.*, 1982). Wherever possible, therefore, the retinas of all apparently unaffected family members were examined. The presence of this phenotype was taken to indicate the presence of the Rb gene. All tumours from enucleated eyes were confirmed histologically as Rb.

Blood samples from all family members were collected either at the clinics in London, through family visits, or via local practitioners who sent patient's samples to the laboratory by first class mail. In our series there were several families in which more than one affected sib was born to

Correspondence: J.K. Cowell.
Received 1 November 1986; and in revised form, 20 January 1987.

unaffected parents but only families in which there were affected members in at least two generations were included in this survey.

*ESD analysis*

Phenotypic expression of the two common electrophoretic variants has been analysed in 50 families. In 9 of these families the analysis is incomplete although in all cases the phenotypes of the affected transmitting members have been determined. In the remaining 41 families the phenotypes of all family members have been determined. Previous analysis of ESD levels in these families (Cowell *et al.*, 1986*a*) showed them all to be within the normal range, thus excluding the possibility of a detectable chromosome deletion. Of the 50 families analysed, 43 were uninformative: in 34 families all individuals had the 1-1 phenotype and in 8 the affected transmitting parent was 1-1 and the unaffected parent was heterozygous. In one family both parents were heterozygotes. Seven families were informative for the polymorphism (Figure 1), and in all cases there was cosegregation of the Rb and the particular ESD allele. In family seven (Figure 1), phase has been established through the daughter in the first marriage. The father and his second wife are currently being counselled for antenatal diagnosis.

**Figure 1** Segregation of the esterase-D electrophoretic variants in seven retinoblastoma families informative for the polymorphism. In each case the first born in the family is shown on the left.. ◐ – Unilateral; ● – Bilateral.

In 6 of the families with an unaffected heterozygous parent the affected children are heterozygotes including one set of twins. These patients will be potentially informative for the ESD polymorphism in the future.

Antenatal diagnosis of Rb using the ESD protein polymorphism, depends on the ability to demonstrate enzyme activity in chorionic villus and cord blood samples. ESD analysis of cord blood samples derived from four patients showing no ocular abnormalities has shown that there is no quantitative or qualitative difference in enzyme activity when compared with adult blood samples. We have also been able to demonstrate that ESD phenotypes can be determined from lysates prepared directly from two chorionic villus samples and in cells from three different short term villus cultures.

*Linkage analysis*

Pedigree analysis was performed using the LIPED programme. The data for each informative family is recorded in Table I. The cumulative lod score was highest at $\theta=0$ with a value of 2.61. In this analysis the penetrance was given as 90%, a figure which seems low from our experience in the UK (unpublished observations). Assessing the penetrance at 95% however made no difference to the results. The allele frequencies were recorded as 0.884 for type-1 and 0.116 for type-2 based on our analysis of over 400 individuals including retinoblastoma patients in the UK as described previously (Cowell *et al.*, 1986*a*). This value is not significantly different from that reported by Harris *et al.* (1974). The lod score of 2.61 is in itself almost sufficient for that required to establish linkage. No recombinants in 45 meioses have been observed in any other published reports (Sparkes *et al.*, 1983, Connolly *et al.*, 1983; Mukai *et al.*, 1984; Halloran *et al.*, 1985) giving a cumulative lod score of 13.69 (Table II)

**Table I** Maximum lod scores from individual families (see **Figure 1**) for linkage between esterase-D and the retinoblastoma locus

| *Family* | *Lod* | $\theta$ |
|---|---|---|
| 1 | 0.55145 | 0.0000 |
| 2 | 0.30103 | 0.0000 |
| 3 | 0.30103 | 0.0000 |
| 4 | 0.30103 | 0.0000 |
| 5 | 0.85773 | 0.0000 |
| 6 | 0.30103 | 0.0000 |
| Total | 2.61 | 0.0000 |

### Discussion

The location of the ESD gene and the gene predisposing to Rb to the same chromosome band suggests the possibility of antenatal diagnosis of Rb using the electrophoretic variants of ESD. Although the two loci are located within the same half of band 13q14 (Cowell *et al.*, 1986*b*) this might represent a large recombination distance. It is difficult to assess the true distance between these two loci because of the relatively low frequency of the type-2 ESD allele in Western populations. The frequency of ESD heterozygotes is much higher in the Japanese population at 0.46 (Horai & Mutsunaga, 1984), so that the possibility of antenatal diagnosis in that population should generally be possible. Despite the low frequency of the 2-allele in the USA, Sparkes *et al.* (1983) were able to demonstrate close linkage using three large pedigrees. From their data alone no recombination was observed from 12 phase-known meioses with a lod score of 3.5. Connolly *et al.* (1983) reported a single large family showing unusually low penetrance of the Rb gene (71%). Unaffected family members transmitting the gene were clearly identifiable. Assuming these individuals to be gene carriers, cosegregation of the disease with the ESD marker was seen in all meioses. Additional reports from Mukai *et al.* (1984) and Halloran *et al.* (1985) also failed to

**Table II** Summed lod scores from the 6 informative families presented in this study compared with the 6 families reported previously

| | | *Recombination fraction* | | | | | |
|---|---|---|---|---|---|---|---|
| *Penetrance* | *Study* | *0.00* | *0.05* | *0.01* | *0.02* | *0.03* | *0.04* |
| 0.9 | this study | 2.61 | 2.31 | 2.01 | 1.41 | 0.85 | 0.30 |
| 0.9 | previous reports | 11.08 | 10.06 | 8.99 | 6.68 | 4.20 | 1.73 |
| | Total | 13.69 | 12.37 | 11.00 | 8.09 | 5.05 | 2.03 |

demonstrate recombination between the ESD and Rb loci in two additional families. The cumulative lod score from all of these reports, including ours, is 13.69.

For accurate counselling of parents following antenatal screening it is important to obtain a reliable estimate of the linkage distance between ESD and the Rb locus. From the available data we estimate that the true recombination fraction is no greater than 6% at the 95% confidence limits, and no greater than 10% at the 99% confidence limits.

Our series of families represents the largest group reported to date. In addition to the low frequency of the rarer allele, we have found that the small size of the families in the UK make risk assessment more difficult. Undoubtedly genetic counselling has had a major influence in these decisions. Before the 1960s, when there was little counselling, families tended to be larger, with more affected members.

Another problem encountered in our series was that in only 3/7 cases did the Rb phenotype segregate with the rare allele; the predictive value of this polymorphism cannot be used in subsequent generations in the other 4 families. On the other hand several families exist where phase has been established and one of the affected children is heterozygous as a result of the 2-allele being introduced by the unaffected parent. In one family the affected child had the 2-2 phenotype which will again be uninformative in future generations.

So far we have been able to track the inheritance of the Rb locus in seven families of whom a number are being counselled for prenatal diagnosis. We have also demonstrated that the ESD phenotypes can be easily demonstrated in fetal blood samples and in chorionic villus samples, thus permitting antenatal diagnosis during the first trimester (Ward *et al.*, 1983; Rodeck *et al.*, 1983). Although this enzyme polymorphism will be invaluable for the families reported here, because of the low frequency of the 2-allele in the UK will restrict widespread use. The recent cloning of the ESD gene (Squire *et al.*, 1986; Lee & Lee, 1986) may allow antenatal diagnosis in other families since the inheritance can be followed using restriction fragment length polymorphisms (see Cowell, 1985). The only informative polymorphism identified to date shows a heterozygosity of 32% (Squire *et al.*, 1986) which should prove more generally useful than the ESD protein polymorphism. Several other probes have also been tested for utility in antenatal diagnosis of Rb; alone none of them were shown to be more closely linked than the ESD gene (Craft personal communication). Using flanking markers Cavenee *et al.* (1986) achieved moderate success in prenatal prediction of Rb in three families.

No recombinants in 45 meioses have been demonstrated between the ESD and Rb loci (including the data from Connolly *et al.*, 1983) suggests close proximity of the ESD and the Rb locus. Recently a candidate for the Rb gene has been isolated (Friend *et al.*, 1986). Even with the availability of the Rb gene, however, antenatal diagnosis may still require a combination of closely linked probes since some families will be informative for only one probe. This principle has been demonstrated with haemophilia A where, despite the isolation of the factor VIII gene, the use of flanking probes is essential for effective widespread antenatal diagonsis (M.E. Pembrey, personal communication).

The ESD electrophoretic polymorphism is still the easiest and most reliable of all of the available tests with results available within 12–16 hours of the sample being taken. It is expected that, with the eventual characterisation of the Rb locus, specific genetic changes leading to the predisposition will be identified in a manner similar to that already demonstrated for thalassaemia (e.g. Old *et al.*, 1986).

We would like to thank Prof. B. Jay, Dr. M. Pembrey, Dr C. Mitchell and Dr J. Pritchard for their critical reading of the manuscript. Our particular thanks go to the many medical practitioners throughout the UK who have assisted in the collection of blood samples. The fetal blood samples were kindly supplied by Dr C. Rodeck at King's College, London, and the chorionic villus samples from Dr D. Rooney, St. Mary's Hospital, Paddington. We are especially grateful to Dr R. Winter for his advice about the linkage analysis.

### References

CAVENEE, W.K., MURPHREE, A.L., SCHULL, M.M., BENEDICT, W.F., SPARKES, R.S., KOCK, E. & NORDENSKJOLD, N. (1986). Prediction of familial predisposition to retinoblastoma. *New Engl. J. Med.*, **314**, 1201.

CONNOLLY, M.J., PAYNE, R.H., JOHNSON, G., GALLIE, B.L., ALLERDICE, P.W., MARSHALL, W.H. & LAWTON, R.D. (1983). Familial, EsD-linked, retinoblastoma with reduced pentrance and variable expressivity. *Hum. Genet.* **65**, 122.

COWELL, J.K. (1985). Tracking the cancer genes in paediatric predisposition syndromes; opportunities for prenatal diagnosis. *Cancer Surveys*, **3**, 573.

COWELL, J.K., RUTLAND, P., JAY, M. & HUNGERFORD, J. (1986*a*). Deletions of the esterase D locus from a survey of 200 retinoblastoma patients. *Hum. Genet.* **72**, 164.

COWELL, J.K., THOMPSON, E. & RUTLAND, P. (1986*b*). The need to screen all retinoblastoma patients for esterase-D activity; detection of submicroscopic deletions. *Arch. Dis. Child.* **62**, 11.

COWELL, J.K., HUNGERFORD, J., RUTLAND, P. & JAY, M. (1986*c*). A chromosomal breakpoint which separates the esterase-D and retinoblastoma predisposition loci in a patient with del(13) (q14–q31). *Cancer Genet. Cytogenet.* (In press).

COWELL, J.K., RUTLAND, P., JAY, M. & HUNGERFORD, J. (1986*d*). Effect of the esterase-D phenotype on its *in vitro* activity. *Hum. Genet.* **74**, 298.

FRIEND, S.H., BERNARDS, R., ROGELJ, S., WEINBERG, R.A., RAPAPORT, J.M., ALBERT, D.M. & DRYJA, T.P. (1986). A human DNA segment with properties of the gene that predisposes to retinoblastoma and osteosarcoma. *Nature*, **323**, 643.

GALLIE, B.L., ELLSWORTH, R.M., ABRAMSON, D.H. & PHILLIPS, R.A. (1982). Retinoblastoma: Spontaneous regression of retinoblastoma or benign manifestation of the mutation? *Br. J. Cancer*, **45**, 513.

HALLORAN, S.L., BOUGHMAN, J.A., DRYJA, T.P. & 4 others (1985). Accuracy of the detection of the retinoblastoma gene by esterase-D linkage. *Arch. Ophthalmol.* **103**, 1329.

HARRIS, H., HOPKINSON, D.A. & ROBSON, E.B. (1974). The incidence of reare alleles determining electrophoretic variants: data on 43 enzyme loci in man. *Ann. Hum. Genet.*, **37**, 237.

HOPKINSON, D.A., MESTRINER, M.A., CORTNER, J. & HARRIS, H. (1973). Esterase-D: a new human polymorphism. *Ann. Hum. Genet.*, **37**, 119.

HORAI, S. & MATSUNAGE, E. (1984). Differential enzyme activities in human esterase D phenotypes. *Hum. Genet.* **66**, 168.

LEE, E.-HP. & LEE, W.H. (1986). Molecular cloning of the human esterase D gene, a genetic marker of retinoblastoma. *Proc. Natl Acad. Sci.*, **83**, 6337.

MUKAI, S., RAPAPORT, J.M., SHIELDS, J.A., AUGSBURGER, J.J. & DRYJA, T.P. (1984). Linkage of genes for human esterase-D and hereditary retinoblastoma. *Am. J. Ophthalmol.*, **97**, 681.

OLD, J.M., HEATH, C., FITCHES, A. & 8 others (1986). First trimester fetal diagnosis for haemoglobinopathies: Report on 200 cases. *Lancet* **ii**, 763.

RODECK, C.H., NICOLAIDES, R.H., MORSMANN, J.M., McKENSIE, C., GOSDEN, C.M. & GOSDEN, J.R. (1983). A single operator technique for first trimester chorion biopsy. *Lancet* **ii**, 1340.

SQUIRE, J., DRYJA, T.P., DUNN, J. & 7 others (1986). Cloning of the esterase D gene; A polymorphic gene probe closely linked to the retinoblastoma locus on chromosome 13. *Proc. Natl Acad Sci.*, **83**, 6573.

SPARKES, R.S., SPARKES, M.C., WILSON, M.G. & 4 others (1980). Regional assignment of genes for human esterase D and retinoblastoma to chromosome band 13q14. *Science*, **208**, 1042.

SPARKES, R.S., MURPHREE, A.L., LINGUA, R.W. & 4 others (1983). Gene for hereditary retinoblastoma assigned to human chromosome 13 by linkage to esterase-D. *Science,* **217,** 971.

SPARKES, R.S., SPARKES, M.C., KALINA, R.E., PAGON, R.A., SALK, D.J. & DISTECHE, C.M. (1984). Separation of the retinoblastoma and esterase D loci in a patient with sporadic retinoblastoma and del (13)(q14.1q22.3). *Human Genetics,* **68,** 258.

SPARKES, R.S. (1985). The genetics of retiboblastoma. *Biochim. Biophys, Acta.,* **780,** 95.

WARD, P., PACKMAN, S., LOUGHMAN, W. & 5 others (1984). Location of the retinoblastoma susceptibility gene(s) and the human esterase-D locus. *J. Med. Genet.,* **21,** 92.

WARD, R.T.H., MODELL, B., PETROU, M., KARAGOZUI, F. & DOURATSOS, E. (1983). Method for sampling chorionic villi in first trimester of pregnancy under guidance of real time ultrasound. *Br. Med. J.,* **286,** 1542.

YUNIS, J.J. & RAMSAY, N. (1978). Retinoblastoma and subband deletion of chromosome 13. *Am J. Dis. Child.,* **132,** 161.